# Regional disparities and influencing factors of high quality medical resources distribution in China

**DOI:** 10.1186/s12939-023-01825-6

**Published:** 2023-01-10

**Authors:** Lei Yuan, Jing Cao, Dong Wang, Dan Yu, Ge Liu, Zhaoxin Qian

**Affiliations:** 1 Xiangya Hospital, Central South University, Changsha, Hunan China; 2 National Clinical Research Center for Geriatric Disorders, Xiangya Hospital, Changsha, Hunan China; 3 Department of Cardiovascular Medicine, Third Xiangya Hospital, Central South University, Changsha, Hunan China

**Keywords:** High quality medical resources, Regional disparities, Inequity, Geographical detector

## Abstract

**Background:**

With the gradual increase of residents’ income and the continuous improvement of medical security system, people’s demand for pursuing higher quality and better medical and health services has been released. However, so far little research has been published on China's high quality medical resources (HQMR). This study aims to understand the spatiotemporal variation trend of HQMR from 2006 to 2020, analyze regional disparity of HQMR in 2020, and further explore the main factors influencing the distribution of HQMR in China.

**Methods:**

The study selected Class III level A hospitals (the highest level medical institutions in China) to represent HQMR. Descriptive statistical methods were used to address the changes in the distribution of HQMR from 2006 to 2020. Lorentz curve, Gini coefficient (G), Theil index (T) and High-quality health resource density index (HHRDI) were used to calculate the degree of inequity. The geographical detector method was used to reveal the key factors influencing the distribution of HQMR.

**Results:**

The total amount of HQMR in China had increased year by year, from 647 Class III level A hospitals in 2006 to 1580 in 2020. In 2020, G for HQMR by population was 0.166, while by geographic area was 0.614. T was consistent with the results for G, and intra-regional contribution rates were higher than inter-regional contribution rates. HHRDI showed that Beijing, Shanghai, and Tianjin had the highest allocated amounts of HQMR. The results of the geographical detector showed that total health costs, government health expenditure, size of resident populations, GDP, number of medical colleges had a significant impact on the spatial distribution of HQMR and the q values were 0.813, 0.781, 0.719, 0.661, 0.492 respectively. There was an interaction between the influencing factors.

**Conclusions:**

China's total HQMR is growing rapidly but is relatively inadequate. The distribution of HQMR by population is better than by geography, and the distribution by geography is less equitable. Population size and geographical area both need to be taken into account when formulating policies, rather than simply increasing the number of HQMR.

## Introduction

Health, as a basic human demand, is the basis for achieving comprehensive human development [1]. Medical resources are an important part of public services, and the equitable allocation of medical resources not only affects the health level of residents, but is also closely related to the healthy and sustainable development of human society [2]. The World Health Organization points out that equity in health services means that members of society should have demand-oriented access to health services, rather than depending on factors such as ethnicity, social status, income level, and religious beliefs [3]. However, inequitable allocation of health resources is currently a global problem, especially in developing countries [4]. In the 2030 Agenda for Sustainable Development, the United Nations has clearly identified "ensuring universal access to health and health care services and achieving universal health coverage" as the main goal.

Since the founding of the People’s Republic of China, China's medical and health care has made great progress. However, the imbalance of medical services between urban and rural areas and among different regions is still very prominent [3, 5–7]. In order to solve the problem of difficulties and high expenses in medical care, China launched a new health care system in 2009, with the goal of "basic medical services for all" and the concept of "providing the basic medical and health system to all people as a public product" [8, 9]. Strengthening the primary care system is a priority of new medical reform in China. The capacity of service delivery at primary care institutions has seen a significant improvement, and the infrastructure of community health centers in cities and township health centers in rural areas has been greatly optimized. Almost every rural town has at least one primary health care facility [10].

With the gradual increase of residents' income and the continuous improvement of the medical insurance system, primary health care services can no longer fully meet the needs of Chinese people, and people's demand for pursuing higher quality and better medical and health services has been released [11]. Due to the uneven distribution of HQMR, people must move across regions to access higher-quality medical and health services. This further exacerbates the social problems of difficulty and high cost of getting medical treatment [12]. Thus, the Chinese government released the "Health China 2030 Plan" in 2016, which proposed to "basically achieve a balanced allocation of high-quality medical and health resources" [13]. In March 2021, China's “Outline of the 14th Five-Year Plan (2021–2025) for National Economic and Social Development and the Long-Range Objectives Through the Year 2035” proposed to accelerate the expansion of HQMR and a balanced regional layout of such resources among different regions in China [14].

HQMR refers to the resources with high quality in the whole medical service system, which is characterized with advanced medical techniques, good medical facilities and service, and standardized management. Based on the current Chinese health care policy, hospitals in China are divided into three classes, and each class is divided into level A, B and C [15]. Class III level A hospitals are the highest level medical institutions in China's current medical service system, with better medical service and management, medical quality and safety, technical level and efficiency. Therefore, this study selects Class III level A hospitals (high-level hospitals) as the study subjects to represent HQMR.

At present, the research on medical fairness theory has made great progress and formed a series of theories such as utilitarian ethical doctrine, egalitarian distribution theory, radical liberal theory, and communitarian theory [16]. A lot of research has been conducted in the fields of medical services and health, accessibility of medical facilities, inequity in medical services, and distribution of medical resources and their influencing factors [17, 18]. An Iranian study assessed geographical distribution of hospitals and inequality of hospital beds against socioeconomic status of residents of five metropolitan cities [19]. A study in Mongolia compared urban and rural areas using the Mann–Whitney U test and further investigated the distribution equality of physicians, nurses, and hospital beds throughout Mongolia using the Gini coefficient [20]. Huimin Yu et al. analyzed from the perspective of distribution by both population and service area the equity of physician locations in 31 provincial administrative regions in China [21]. Baoguo Shi et al. explored the allocation patterns of elite hospitals in China and their influencing factors using a linear regression model [11]. Yue Zhang et al. analyzed equity and efficiency of primary health care resource allocation in mainland China by the Lorenz curve, Gini coefficient, Theil index and health resource density index [22]. In addition, there are also studies that analyzed differences and inequalities in regional distribution of health resources in selected Chinese provinces [23–26].

In general, most studies have focused on the regional differences and equity of overall or basic medical resources, and there are fewer studies related to the regional differences and influencing factors of HQMR, and there are no studies on the effects of interactions between different influencing factors on the distribution of HQMR. Therefore, this study assesses the equity of the current allocation of HQMR in China by analyzing the regional differences and influencing factors of HQMR in China, so as to provide reference of the HQMR allocation for China and other regions.

## Methods

### Data source

Data on the provincial high-level hospitals for the years 2006 to 2020 and factors affecting the distribution of HQMR were obtained either from the China Health Statistical Yearbook published by the National Health Commission or the China Statistics Yearbook published by the National Bureau of Statistics.

The layout of HQMR is influenced by diverse and complex factors including demographic factors, medical insurance, education level, economic development, and medical expenditures. The indicators that characterize demographic factors include size of resident populations, population density, and proportion of urban population in total population. The indicators that medical insurance include medical insurance density, and medical insurance depth. The indicators of education level include the proportion of population with college degree and above in the total population and the number of medical colleges. The indicators that characterize economic development include GDP and disposable income. The indicators reflecting medical expenditures include residents' health care expenditure, total health costs and government health expenditure. The details are shown in Table 1.

**Table 1:** Explanatory variables in the study

Factor	Specific indicator	Unit	Factor code
Demographic factors	Size of resident populations	10,000 persons	x_1_
	Population density	person	x_2_
	Proportion of urban population in total population	%	x_3_
Medical insurance	Medical insurance density	CNY/person	x_4_
	Medical insurance depth	%	x_5_
Education level	Proportion of population with college degree and above in the total population	%	x_6_
	Number of medical colleges	/	x_7_
Economic Development	GDP	100 million CNY	x_8_
	Disposable income	CNY	x_9_
Medical expenditures	Residents' health care expenditure	CNY	x_10_
	Total health costs	100 million CNY	x_11_
	Government health expenditure	100 million CNY	x_12_

*CNY* Chinese yuan, *GDP* Gross Domestic Product

### Setting

**Fig. 1:**
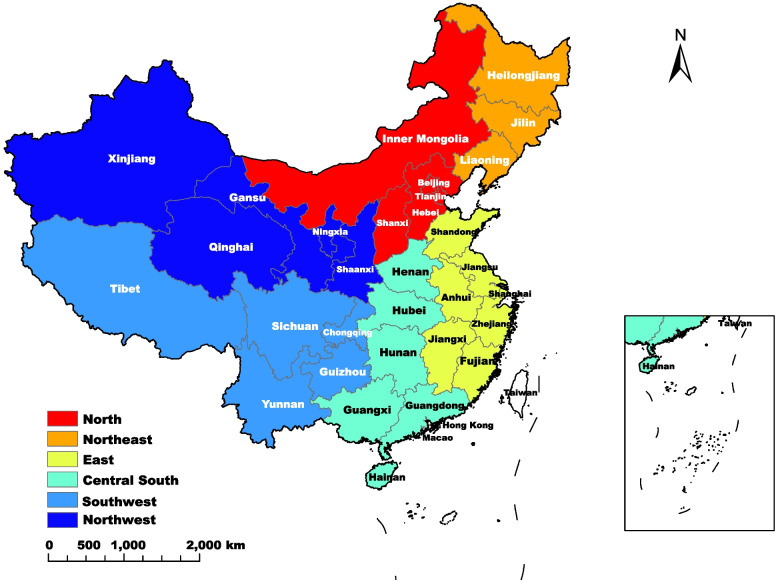
The distribution of 6 administrative regions in China

China's land area is approximately 9.6 million square kilometers and is divided into 34 provincial administrative regions. This study selected 31 provinces in mainland China except for Hong Kong special administrative region, Macao special administrative region, and Taiwan province due to inconsistency of statistics caliber and data collection. According to economic factors and geographical locations, 31 provinces are divided into 6 administrative regions as follows, North: including Beijing, Tianjin, Hebei, Shanxi, Inner Mongolia. Northeast: including Heilongjiang, Jilin, Liaoning. East: including Shanghai, Jiangsu, Zhejiang, Anhui, Fujian, Jiangxi, Shandong. Central South: including Henan, Hubei, Hunan, Guangdong, Guangxi, Hainan. Southwest: including Chongqing, Sichuan, Guizhou, Yunnan, Tibet. Northwest: including Shaanxi, Gansu, Qinghai, Ningxia, Xinjiang. The geographical locations of 6 administrative regions are shown in Fig. 1.

### Data analysis

Descriptive statistical methods were used to address changes in the distribution of HQMR from 2006 to 2020. The ArcGIS 10.5 software was used to draw the distribution map of China's HQMR in 2006, 2011, 2016, and 2020. The allocation inequity of high-level hospitals in 2020 based on both population and geographical distribution was calculated by the Lorentz curve, Gini coefficient, Theil index and High-quality health resource density index. The geographical detector method was used to analyze the key factors influencing the distribution of HQMR.

### The Lorenz curve

The Lorenz curve is a common tool to evaluate the equity of health and medical resources allocation in the field of public health [27]. The bending degree of the Lorenz curve can reflect the inequality of resource allocation. A 45° line indicates absolute equity. If the Lorenz Curve is closer to the absolute equity line, the allocation of health resources is more equitable [28]. In this study, the number of high-level hospitals per capita (area) was in ascending order, with the X-axis representing the cumulative percentage of the population or area, and the Y-axis representing the cumulative percentage of the high-level hospitals.

### The Gini coefficient (G)

G is usually used to assess the equity of income and resource allocation, which was derived from the Lorenz curve [29, 30]. G ranges from 0 to 1. “0” means the evenest distribution of medical and health resources while “1” means the most concentrated and inequitable. 0 < G < 0.2 indicates that the distribution of medical resources is of absolute equity; 0.2 ≤ G < 0.3, relative equity; 0.3 ≤ G < 0.4, proper equity; 0.4 ≤ G < 0.5, relative inequity; 0.5 ≤ G < 1 severe inequity. The formula of the G is as follows.

$$G=1-\sum\limits_{i=1}^{n}\left({X}_{i+1}-{X}_{i}\right)\left({Y}_{i+1}+{Y}_{i}\right)$$

In the formula, ${X}_{i}$ illustrates the cumulative percentage of the population and area in the ith district., and ${Y}_{i}$ illustrates the cumulative percentage of high-level hospitals in the ith district. n is the number of 31 provinces.

### Theil Index (T)

T can be used to analyze the source of inequity. The advantage of T is that it measures the contribution of both intra- and inter-regional differences to overall inequality. T ranges from 0 to 1. Generally, the smaller the T value, the more balanced the resource distribution. T calculated the formula as follows:

$$T=\sum\limits_{i=1}^{n}{P}_{i}\times log\left(\frac{\overline{\mathrm{R}} }{{\overline{R} }_{i}}\right)$$

In the formula, ${P}_{i}$ is the proportion of every province’s population (area); R is a high-quality hospital allocated by population (area) in the 31 provinces; and ${R}_{i}$ is the total population (area) in the 31 provinces nationwide. n is the number of 31 provinces.

G and T were calculated based on population and area in this study.

*T* can be divided into ${T}_{inter}$ and ${T}_{intra}$, and the calculation of ${T}_{inter}$ and ${T}_{intra}$ is as follows [31]:

$$T={T}_{inter}{ + T}_{intra}$$

$${T}_{inter}=\sum\limits_{j=1}^{m}{P}_{j}\times log\left(\frac{{P}_{j}}{{Y}_{j}}\right)$$

$${T}_{intra}=\sum\limits_{j=1}^{m}{P}_{j}\times {T}_{j}$$

${P}_{j}$: proportion of the six groups’ (North, Northeast, East, Central South, Southwest and Northwest regions) population (area) accounting for the overall population of China.

${Y}_{j}$: proportion of high-level hospitals owned by the six groups (North, Northeast, East, Central South, Southwest and Northwest regions) accounting for the total number of high-level hospitals nationwide.

${T}_{j}$: T of the six groups (North, Northeast, East, Central South, Southwest and Northwest regions).

The contribution rate of intra- and inter-region can be calculated by dividing ${T}_{intra}$ /T and ${T}_{inter}$ /T [32].

### High-quality Health Resource Density Index (HHRDI)

The health resource density index comprehensively considers the influencing factors of population and geographical area, and can better reflect the comprehensive level of the distribution of health resources by population and geographical area [22]. Therefore, this study refers to the calculation principle of the health resource density index to establish a high-quality health resource density index (HHRDI). The calculation formula is:

$$\mathrm{HHRDI}=\frac{{HHR}_{i}}{\sqrt{{A}_{i}\times {P}_{i}}}$$

In the formula, $HH{R}_{i}$: HQMR quantity of the ith region. ${A}_{i}$: geography of the ith region. ${P}_{i}$: population of the ith region.

### Geographical Detector

The geographical detector is a set of statistical methods that detect spatial heterogeneity and reveal the driving forces behind it [33]. The geographically weighted regression is a linear model, while the geographical detector is a nonlinear model. The advantage of the geographical detector is that it can quantify the interaction force between two independent variables and two dependent variables without considering multicollinearity [34]. The geographical detector is widely used to explore the formation mechanism of the spatial distribution of geographic objects, including risk detection, factor detection, ecological detection, and interactive detection [35].

Factor detection and interactive detection methods were used in this study. The factor detection mainly measures the influence of each factor on the HQMR; the interactive detection mainly analyzes the influence of the interaction between the factors on the distribution of HQMR, that is, the combined effect of the two factors—whether it will increase or decrease the influence on HQMR. The factor detection is calculated as follows:

$$q=1-\frac{\sum_{m=1}^{L}{{\sigma }_{m}^{2}N}_{m}}{\mathrm{N}{\sigma }^{2}}$$

In the formula, the value range of q is [0, 1] and the larger the q value, the stronger the explanatory power of the independent variable X to the attribute dependent variable Y; m = 1, …; L: the stratification of the factor X and the variable Y; Nm and N: the number of units in the layer m and the whole area, respectively; ${\sigma }_{m}^{2}$ and ${\sigma }^{2}$: the layer m and the variance of the Y values for the whole area respectively.

Interaction detection is used to detect whether the interaction of two influences enhances, weakens, or is independent in explaining the spatial variation on the dependent variable. It is discriminated by comparing the magnitude of q(X_1_), q(X_2_) and q(X_1_ ∩ X_2_). The specific classification is shown in Table 2.

**Table 2:** The types of factor interaction expression

Expression	Interaction
q(x_1_ ∩ x_2_) < Min(q(x_1_), q(x_2_))	Weaken, nonlinear
Min(q(x_1_), q(x_2_)) < q(x_1_ ∩ x_2_) < Max(q(x_1_), q(x_2_))	Weaken, univariate
q(x_1_ ∩ x_2_) > Max(q(x_1_), q(x_2_))	Enhance, bivariate
q(x_1_ ∩ x_2_) = q(x_1_) + q(x_2_)	Independent
q(x_1_ ∩ x_2_) > q(x_1_) + q(x_2_)	Enhance, nonlinear

As geographic detectors are applied to the characteristics of the type variables, the potential influence factor data were first discretized separately using the natural breakpoint grading function of ArcGIS software and classified into five categories in order of their values from highest to lowest. The geographical detector software platform compiled by excel was used to perform the analyses. *p* < 0.05 was considered statistically significant.

## Results

### Spatiotemporal variation of HQMR

As shown in Table 3, the total amount of HQMR in China had been increasing rapidly in recent years. The number of high-level hospitals increased from 647 in 2006 to 1,580 in 2020; the growth rates (GRs) was 144.2%, and the average annual increase was 62.2 hospitals. The growth rates of Southwest, Northwest, and East were higher than the national level, and GRs from 2006 to 2020 were 314.29%, 206.67%, and 181.82%, respectively.

**Table 3:** Quantity of HQMR in China from 2006 to 2020

Region	Province	2006	2011	2016	2020	GRs
North		118	135	193	222	88.14%
	Beijing	37	37	54	55	48.65%
	Tianjin	21	22	31	31	47.62%
	Hebei	32	35	44	52	62.50%
	Shanxi	17	27	41	43	152.94%
	Inner Mongolia	11	14	23	41	272.73%
Northeast		88	111	158	168	90.91%
	Liaoning	40	46	64	65	62.50%
	Jilin	19	22	29	30	57.89%
	Heilongjiang	29	43	65	73	151.72%
East		154	259	373	434	181.82%
	Shanghai	24	29	32	32	33.33%
	Jiangsu	39	52	69	82	110.26%
	Zhejiang	22	46	70	67	204.55%
	Anhui	14	27	41	52	271.43%
	Fujian	5	26	34	40	700.00%
	Jiangxi	12	40	44	53	341.67%
	Shandong	38	39	83	108	184.21%
Central South		186	237	327	386	107.53%
	Henan	29	39	51	68	134.48%
	Hubei	45	50	72	73	62.22%
	Hunan	25	32	43	56	124.00%
	Guangdong	54	70	108	122	125.93%
	Guangxi	28	40	43	51	82.14%
	Hainan	5	6	10	16	220.00%
Southwest		56	75	159	232	314.29%
	Chongqing	14	13	27	33	135.71%
	Sichuan	23	41	67	105	356.52%
	Guizhou	9	9	29	31	244.44%
	Yunnan	9	10	34	54	500.00%
	Tibet	1	2	2	9	800.00%
Northwest		45	64	98	138	206.67%
	Shaanxi	18	29	34	45	150.00%
	Gansu	6	12	17	30	400.00%
	Qinghai	6	8	10	10	66.67%
	Ningxia	3	3	6	6	100.00%
	Xinjiang	12	12	31	47	291.67%
Nationwide		647	881	1308	1580	144.20%

*HQMR* High quality medical resources, *GRs* the growth rates

**Fig. 2:**
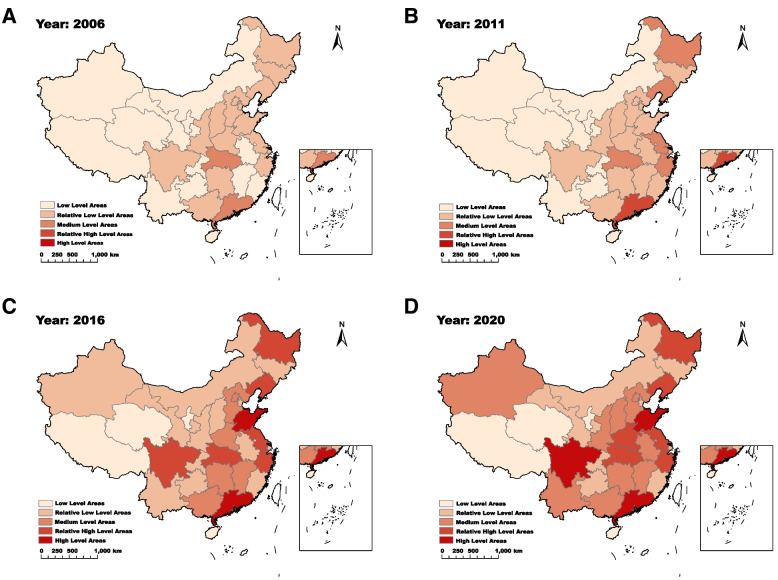
Spatial distribution of HQMR changes in China. **A** distribution of high-level hospitals in China in 2006; **B** distribution of high-level hospitals in China in 2011; **C** distribution of high-level hospitals in China in 2016; **D** distribution of high-level hospitals in China in 2020

Based on the provincial high-level hospitals data in 2006, 2011, 2016, and 2020, the Natural Breaks method was used to divide the 31 provincial high-level hospitals into five categories, namely, Low Level Areas (1–16), Relative Low Level Areas (17–41), Medium Level Areas (42–56), Relative High Level Areas (57–82) and High-Level Areas (83–122). As shown in Fig. 2, from 2006 to 2020, the supply level of HQMR in China increased rapidly; the number of Low Level Areas decreased from 14 provinces to three provinces; the number of Relative Low Level areas decreased from 16 provinces to eight provinces; the number of Medium Level areas increased from two provinces to ten provinces; six provinces developed into Relative High Level Areas, and three provinces including Guangdong, Sichuan, and Shandong entered High Level Areas.

### HQMR distribution in 2020 in China

In 2020, there were 1,580 high-level hospitals in China, with an average of 51 in each province. From a regional perspective, the number of high-level hospitals in East (434) and Central South (386) regions was higher than that in other regions, followed by Southwest (232), North (222), Northeast (168), and Northwest (138). From the perspective of provinces, Guangdong, Shandong, and Sichuan have more than 100 high-level hospitals, 122, 108, and 105 respectively. The number of high-level hospitals of 15 provinces is lower than the national average, and Tibet and Ningxia have the least, 9 and 6 respectively. See Table 4 for details.

**Table 4:** Distribution of HQMR in China in 2020

Region	Province	Population (10,000 persons)	Total area (km^2^)	High-level hospitals	High-level hospitals		HHRDI
					Per million persons	Per 10,000 km^2^	
North		16,933	1,561,881	222	1.31	1.42	1.37
	Beijing	2189	16,418	55	2.51	33.50	9.17
	Tianjin	1387	11,934	31	2.24	25.98	7.62
	Hebei	7464	187,185	52	0.70	2.78	1.39
	Shanxi	3490	156,698	43	1.23	2.74	1.84
	Inner Mongolia	2403	1,189,646	41	1.71	0.34	0.77
Northeast		9825	778,083	168	1.71	2.16	1.92
	Liaoning	4255	148,121	65	1.53	4.39	2.59
	Jilin	2399	190,234	30	1.25	1.58	1.40
	Heilongjiang	3171	439,728	73	2.30	1.66	1.95
East		42,383	802,463	434	1.02	5.41	2.35
	Shanghai	2488	6340	32	1.29	50.47	8.06
	Jiangsu	8477	102,248	82	0.97	8.02	2.79
	Zhejiang	6468	103,617	67	1.04	6.47	2.59
	Anhui	6105	139,968	52	0.85	3.72	1.78
	Fujian	4161	123,467	40	0.96	3.24	1.76
	Jiangxi	4519	167,935	53	1.17	3.16	1.92
	Shandong	10,165	158,888	108	1.06	6.80	2.69
Central South		40,986	1,015,480	386	0.94	3.80	1.89
	Henan	9941	165,662	68	0.68	4.10	1.68
	Hubei	5745	186,166	73	1.27	3.92	2.23
	Hunan	6645	211,833	56	0.84	2.64	1.49
	Guangdong	12,624	179,645	122	0.97	6.79	2.56
	Guangxi	5019	237,804	51	1.02	2.14	1.48
	Hainan	1012	34,370	16	1.58	4.66	2.71
Southwest		20,526	2,333,491	232	1.13	0.99	1.06
	Chongqing	3209	82,370	33	1.03	4.01	2.03
	Sichuan	8371	491,458	105	1.25	2.14	1.64
	Guizhou	3858	176,179	31	0.80	1.76	1.19
	Yunnan	4722	389,437	54	1.14	1.39	1.26
	Tibet	366	1,194,047	9	2.46	0.08	0.43
Northwest		10,360	3,091,315	138	1.33	0.45	0.77
	Shaanxi	3955	205,639	45	1.14	2.19	1.58
	Gansu	2501	457,793	30	1.20	0.66	0.89
	Qinghai	593	696,583	10	1.69	0.14	0.49
	Ningxia	721	66,400	6	0.83	0.90	0.87
	Xinjiang	2590	1,664,900	47	1.81	0.28	0.72
Nationwide		141,013	9,582,713	1580	1.12	1.65	1.36

*HQMR* High quality medical resources, *HHRDI* High-quality health resource density index

The national average number of high-level hospitals per million population in 2020 was 1.12. From a regional perspective, the number of high-level hospitals per million population of Northeast was the highest (1.71), followed by Northwest (1.33), North (1.31), and Southwest (1.13), while East and Central South regions had 1.02 and 0.94 respectively, lower than the national average. In terms of provinces, Beijing has the most high-level hospitals (2.51), whereas Henan province has the least (0.68). The number of high-level hospitals per million population of 13 provinces including Henan, Hebei, Guizhou, and Ningxia was lower than the national average.

The national average number of high-level hospitals per 10,000 km^2^ in 2020 was 1.65. From a regional perspective, the number of high-level hospitals per 10,000 km^2^ of East was the highest (5.41), followed by Central South (3.80) and Northeast (2.16), while North (1.42), Southwest (0.99), and Northwest (0.45) were lower than the national average. From the perspective of provinces, the top five provinces in terms of the number of high-level hospitals per 10,000 km^2^ were Shanghai, Beijing, Tianjin, Jiangsu, and Shandong, while nine provinces including Inner Mongolia, Xinjiang, Qinghai, and Tibet were lower than the national average.

### Equity of HQMR distribution in China in 2020

#### Equity based on Lorenz curve and G

**Fig. 3:**
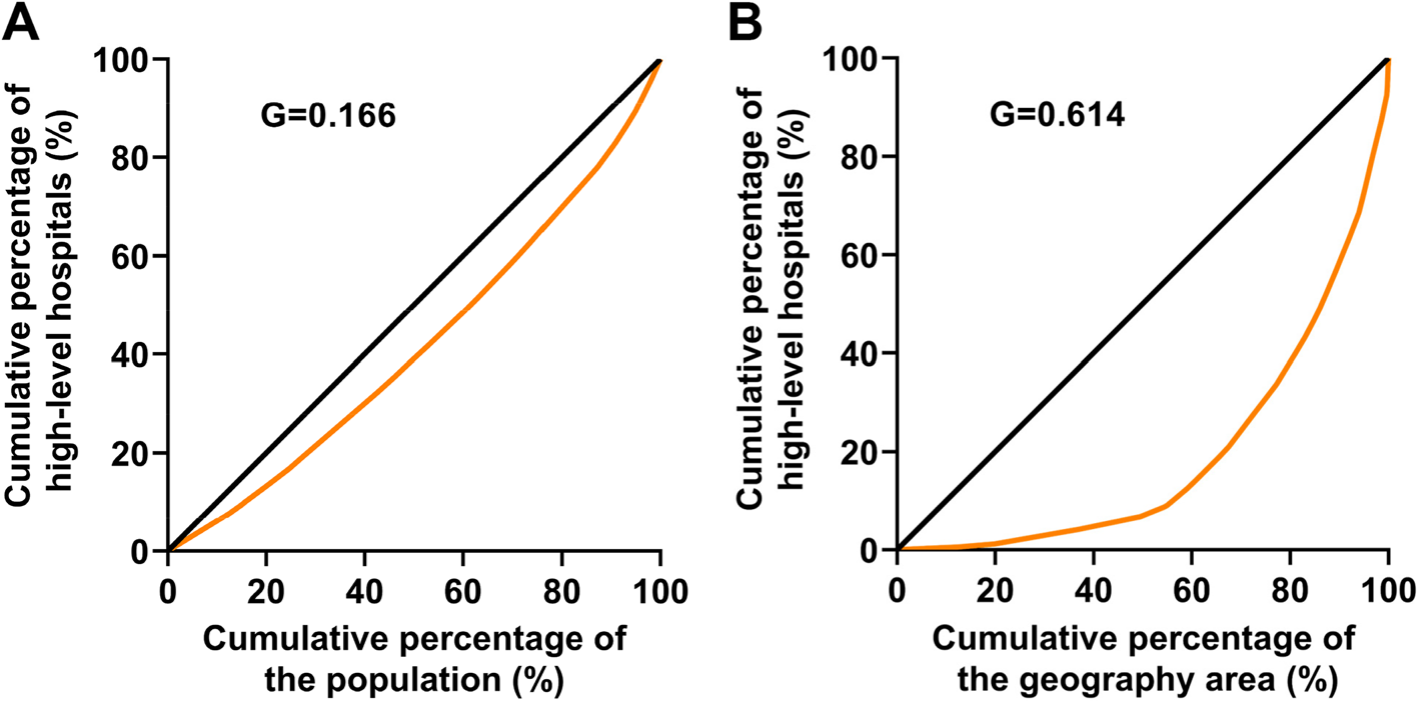
Lorenz curve of the distribution of HQMR. **A** the Lorenz curve by population distribution; **B** The Lorenz curve by geographical distribution

Figure 3 shows that the Lorenz curve by population distribution was close to the absolute equity line, and the calculated G was 0.166, indicating that HQMR was in an absolutely fair state by population in 2020. The Lorenz curve by geographical area was far from the absolute equity line, and the calculated G was 0.614, indicating that the distribution of HQMR by geographical area in 2020 was severely inequal.

**Fig. 4:**
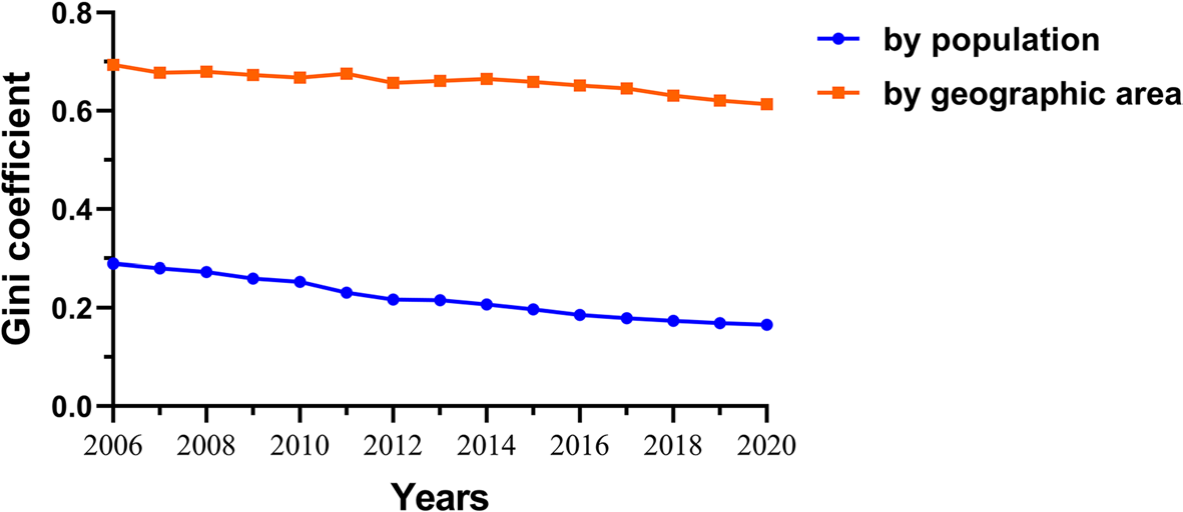
Inequality trends based on the Gini coefficient in China from 2006 to 2020

To see whether the inequality of the HQMR distribution has improved, we proceeded with a longitudinal analysis of changes in inequality over time. Figure 4 shows the change in the G of China’s HQMR from 2006 to 2020. The G by population dropped from 0.290 in 2006 to 0.166 in 2020, which means that the distribution of HQMR by population had improved from a state of relative equity to absolute equity. The G by geographic area decreased from 0.694 in 2006 to 0.614 in 2020, showing an overall decreasing trend and suggesting a certain degree of improvement in HQMR equity. However, the G were all greater than 0.6, indicating that the distribution of HQMR by geographic area was still in a state of severe inequity.

**Fig. 5:**
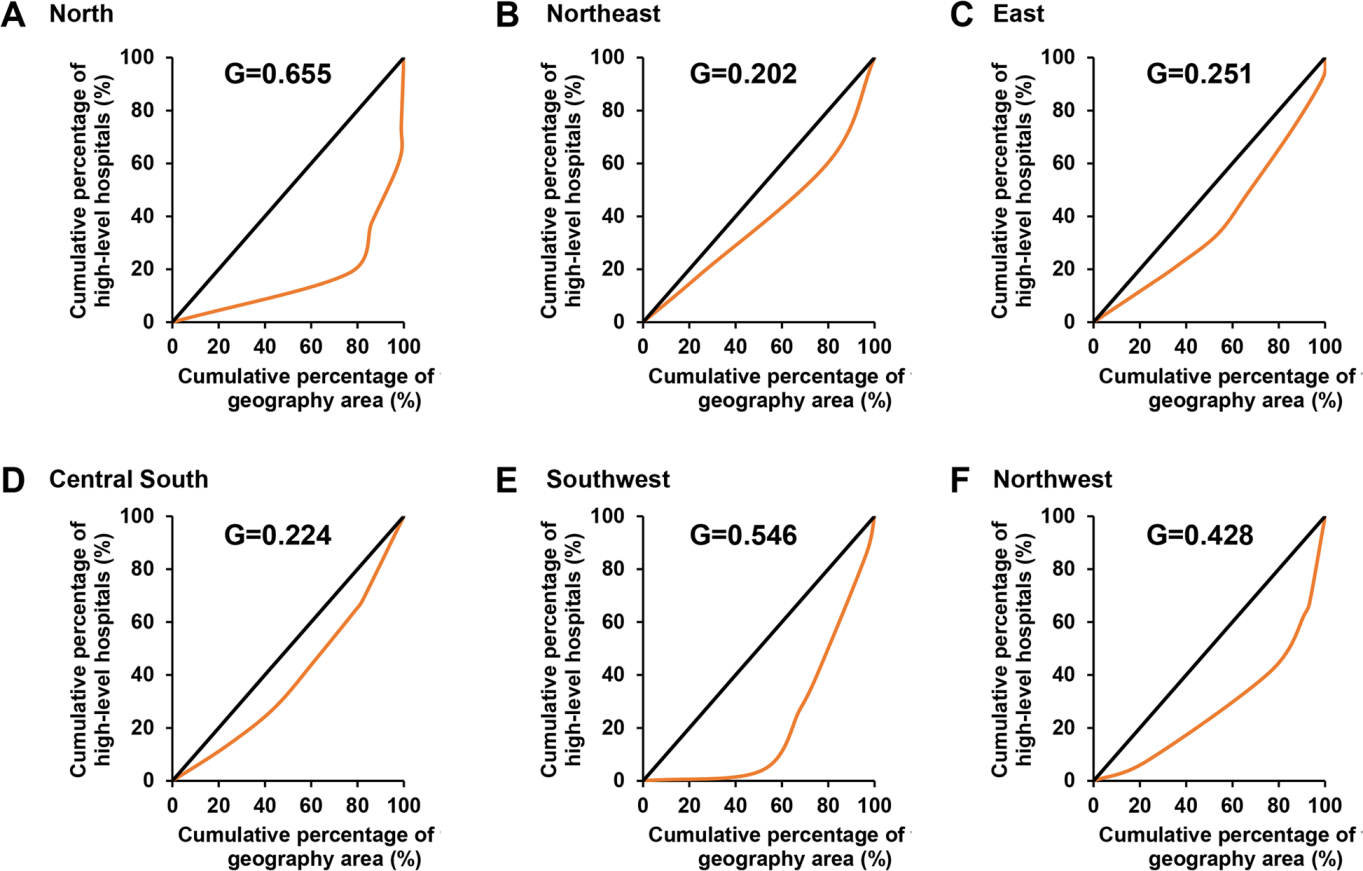
Lorenz curves and Gini coefficients of HQMR by geographic area in different regions in 2020. **A** Lorenz curves and Gini coefficients of HQMR by geographic area in North in 2020; **B** Lorenz curves and Gini coefficients of HQMR by geographic area in Northeast in 2020; **C** Lorenz curves and Gini coefficients of HQMR by geographic area in East in 2020; **D** Lorenz curves and Gini coefficients of HQMR by geographic area in Central South in 2020; **E** Lorenz curves and Gini coefficients of HQMR by geographic area in Southwest in 2020; **F** Lorenz curves and Gini coefficients of HQMR by geographic area in Northwest in 2020

Figure 5 shows the Lorenz curves and G of the six regions by geographical area. Lorenz curves of Northeast, East, and South Central were relatively close to the absolute equity line, with Gini coefficients of 0.202, 0.251, and 0.224 respectively, which was a relatively equitable state. The Lorenz curves of North, Southwest, and Northwest were far from the absolute equity line, with Gini coefficients of 0.655, 0.546, and 0.428 respectively, which was an inequitable state. It suggests that the geographical inequity of HQMR distribution in China in 2020 may mainly come from North, Southwest, and Northwest.

#### Equity based on T

The T by regional population of HQMR was 0.022, and the contribution rate of inter-and intra-region was 32% and 68% respectively. The T by regional area of HQMR was 0.315, and the contribution rate of inter-and intra-region were 48% and 52% respectively. The T of HQMS showed the same trend with the G and Lorenz curve and the inequality was mainly attributed to intra-regional differences.

#### HHRDI

As shown in Table 4, the national HHRDI in 2020 was 1.36. HHRDI of 23 provinces was higher than the national average. Among them, Beijing, Shanghai, Tianjin were the top three, with 9.17, 8.06, 7.62, respectively. HHRDI of 8 western provinces including Yunnan, Guizhou, Gansu, Ningxia, Inner Mongolia, Xinjiang, Qinghai and Tibet provinces were lower than the national average, with 1.26, 1.19, 0.89, 0.87, 0.77, 0.72, 0.49, 0.43 respectively.

### Analysis of influencing factors

The explanatory power (*q*-value) of each factor on the spatial heterogeneity of HQMR in China in 2020 and the significant *P*-values are shown in Table 5. Based on the q value, the factor detector reveals the extent to which a factor explains the spatial distribution of HQMR. The q values are sorted in the following order: x_11_(0.813) > x_12_(0.781) > x_1_(0.719) > x_8_(0.661) > x_7_(0.492) > x_2_(0.393) > x_9_(0.228) > x_5_(0.181) > x_10_(0.179) > x_3_(0.158) > x_6_(0.055) > x_4_(0.02). Five factors passed the significance test at the 5% level, total health costs (x_11_) and government health expenditure(x_12_), size of resident populations(x_1_), GDP(x_8_), number of medical colleges(x_7_), respectively, indicating that these five factors made significant contribution to the spatial distribution of HQMR, and their explanatory power reached 81.3%,78.1%, 71.9%, 66.1%, 49.2%, respectively.

**Table 5:** Results of the factor detection

	x_1_	x_2_	x_3_	x_4_	x_5_	x_6_	x_7_	x_8_	x_9_	x_10_	x_11_	x_12_
q	0.719	0.393	0.158	0.020	0.181	0.055	0.492	0.661	0.228	0.179	0.813	0.781
*p*	0.000	0.109	0.441	0.976	0.463	0.865	0.025	0.009	0.410	0.299	0.000	0.000

The interaction detection results between factors were shown in Table 6. The factors of all interaction types were “Enhance, nonlinear”, indicating that there was an interaction between the influencing factors, and the explanatory power of any two factors interactions was greater than that of a single factor. This means that the distribution of HQMR in China is not caused by a single influencing factor, but results from a combination of different influencing factors. The interaction between medical insurance density (x_4_) and total health costs (x_11_) has the largest impact, which is 0.980. In addition, the phenomenon worthy of attention was that although population density(x_2_), medical insurance density (x_4_), medical insurance depth(x_5_), proportion of population with college degree and above in the total population(x_6_) alone had no significant influence on the distribution of HQMR, they had a large effect on HQMR through its interaction with other factors. Especially the interactive influence of the factor pairs x_6_ ∩ x_11_, x_1_ ∩ x_4_, x_2_ ∩ x_11_, and x_5_ ∩ x_11_ were greater than 0.90, which indicates these four factors cannot be neglected in the development and allocation of HQMR.

**Table 6:** Results of the interactive detection

	x_1_	x_2_	x_3_	x_4_	x_5_	x_6_	x_7_	x_8_	x_9_	x_10_	x_11_	x_12_
x_1_	0.719											
x_2_	0.836	0.393										
x_3_	0.896	0.568	0.158									
x_4_	0.921	0.668	0.355	0.020								
x_5_	0.777	0.498	0.385	0.377	0.181							
x_6_	0.887	0.548	0.277	0.347	0.469	0.055						
x_7_	0.781	0.639	0.782	0.712	0.607	0.796	0.492					
x_8_	0.792	0.805	0.864	0.830	0.833	0.815	0.775	0.661				
x_9_	0.825	0.522	0.633	0.454	0.501	0.548	0.786	0.781	0.228			
x_10_	0.808	0.558	0.336	0.502	0.468	0.392	0.733	0.835	0.506	0.179		
x_11_	0.858	0.920	0.882	0.980	0.913	0.937	0.857	0.841	0.848	0.927	0.813	
x_12_	0.809	0.840	0.840	0.881	0.821	0.908	0.858	0.834	0.861	0.868	0.902	0.781

Overall, the geographic distribution of HQMR in China in 2020 was the result of a combination of factors, of which five factors, including total health costs, government health expenditure, size of resident populations, GDP, and number of medical colleges had a direct and significant impact on the distribution.

## Discussion

This study describes the changes in China's HQMR from 2006 to 2020 and explores regional disparities of HQMR and its determinants in 2020, and summarizes four major findings.

First, the total amount of HQMR in China has increased rapidly in recent years, but the demand for quality resources is also increasing. The results of this study show that between 2006 and 2020, the number of high-level hospitals increased from 647 to 1580 with a growth rate of 144.2%; the number of high-level hospitals per million population increased from 0.49 to 1.12; the average number of high-level hospitals per 10,000 km^2^ increased from 0.68 to 1. 65. The growth rate of total HQMR is higher than the population growth rate, which means that the access to quality medical services for residents is increasing [36]. However, as the residents' economic status and living standards continue to improve and the continuous improvement of the medical insurance system, the people's demand for higher quality and better healthcare services is also rapidly growing. In addition, Chinese residents lack trust in the standard of care in primary health care institutions and are willing to pay more and spend more time seeking care in high-level hospitals [37]. According to data from the 2020 China Health Statistics Yearbook, from 2015 to 2019, the bed utilization rate of tertiary general hospitals affiliated with the National Health Commission increased from 102.1% to 106.3%, and the bed utilization rate of tertiary general hospitals at the provincial level was about 100%, while during the same period the bed utilization rate of secondary and primary hospitals decreased from 84.1% to 81.6% and 58.8% to 54.7%, respectively. The efficiency of medical resource utilization reflects the patients' demand for HQMR from the side.

Second, we found that there are obvious regional differences in the distribution of HQMR in China. Regionally, East and Central South regions have more high-level hospitals, with a number of 434 and 386 respectively, much higher than the other four regions. Other similar studies have also found significant regional differences in China's health care resources, with significantly higher-quality physicians and health care resources in the east than in the west [3, 38, 39]. The three provinces with the highest HHRDI were Beijing, Shanghai, and Tianjin, which were significantly higher than the national average. Due to the richness of HQMR in Beijing and Shanghai, a large number of patients from other parts of China have long been attracted to "cross-regional access to medical care ", with 37.21% and 40.12% of inpatients in tertiary hospitals in Beijing and Shanghai, respectively, coming from outside the region in 2019 [40]. According to the China National Medical Service and Quality Safety Report, the top 5 provinces with the highest outflow ratio in 2019 were Tibet, Anhui, Inner Mongolia, Hebei and Gansu, which are mainly in the central and western regions, with outflow ratios of 27.87%, 18.38%, 17.02%, 14.09% and 11.91%, respectively. The top 5 provinces for patient inflow were Shanghai, Beijing, Jiangsu, Zhejiang and Guangdong, and the inflow provinces were basically concentrated in the eastern regions with developed HQMR. As a result, the problem of difficulty and high cost of getting medical treatment is further exacerbated by the large number of patients chasing HQMR and health services across regions [12, 41].

Third, the inequality of HQMR is mainly reflected in the geographic distribution rather than population. The Lorenz curve and G results of this study show that the allocation of HQMR by population distribution is in an equitable state, and the disparity in the allocation of HQMR by geographic area is huge. The Chinese government has issued numerous documents to optimize the allocation of health resources. When HQMR is limited, these documents recommend prioritizing the allocation of HQMR based on population [31]. Thus, the equity of resource allocation based on population size is significantly better than that based on geographic area. Similar results have been found in other studies [42–44]. Equity in health care services includes not only population equity but also geographic equity [38]. The geographic accessibility of health care services is closely related to population health outcomes [45]. Therefore, it is reasonable to recommend that both demographic and geographic factors should be taken into account when the government makes health planning [46]. At present, health resources especially HQMS should be more allocated to economically underdeveloped and remote regions to gradually improve geographic equity and promote balanced regional development.

Finally, the results of the analysis of influencing factors proved that size of resident populations, number of medical colleges, GDP, total health costs and government health expenditure are the key factors influencing the allocation of HQMR in China. Previous studies also found that the resident population has a positive effect on the allocation of healthcare resources, probably because the larger the size of the resident population, the greater the demand for healthcare services will be, which is one of the factors considered by the government in allocating HQMR [47]. The number of medical colleges is also an important factor in the distribution of HQMR in China. This is mainly because in China, medical colleges usually have one or several affiliated hospitals, which serve as internship sites for medical schools to train clinicians and to absorb outstanding graduates to stay in the hospitals. Therefore, these affiliated hospitals are generally the best hospitals in the region, which in turn creates a concentration of HQMR [11]. GDP is an important indicator of a country's or region's economic status and development level. It is generally believed that the economic development of a region can provide strong support for medical health expenditure. Governments in better-off regions are able to afford to invest in HQMR, while poorer areas cannot. In addition, Liu W et al. also found that cities with better economic conditions are easy to attract HQMR [3]. This may be because regions with better economic conditions have more growth opportunities and higher incomes, which will attract more health professionals to employment. The total health cost is the total monetary amount of health resources of a country or region raised from the whole society to carry out the health service activities in a certain period, which can reflect the degree of emphasis on health care under certain economic conditions and cost burden levels [48]. Due to the high level of medical services provided by high-level hospitals, the price of HQMR services is higher than that of basic medical services, and the cost of medical expenses is also higher. Governments can influence health outcomes in a country or region in many ways, such as by developing health programs and increasing health investments [49]. Huanhuan Jia et al. found that the Chinese government plays a leading role in health and has a crucial influence on health development [50]. In China, government-established public medical institutions dominate the healthcare system, so government health expenditure play a key role in expanding HQMR.

In order to enable the majority of people to enjoy quality medical and health services close to their homes and reduce the phenomenon of cross-regional access to medical care, there are a few suggestions. On the one hand, China needs to continue to expand the total amount of HQMR to solve the problem of "inadequate" development. Measures include continually enhancing the sources of financial investment in the medical and health fields and encouraging social capital to support and participate in medical care, and guiding social forces to improve medical facilities and equipment [40]. On the other hand, it is more important and urgent to accelerate the balanced distribution of HQMR in China. First, China’s government can promote the horizontal flow of HQMR, and guide the layout of HQMR to areas with weak medical service capacity and huge public demand for medical services. For example, the central government ought to select high-level medical institutions from areas rich in HQMR, such as Beijing and Shanghai, and encourage these hospitals to build regional medical centers in areas with high patient outflow and relatively weak medical resources [51]. Furthermore, the local government can introduce experienced and excellent medical personnel to work in economically underdeveloped and remote regions by giving generous subsidies and improving their social status. Second, it is necessary to promote the vertical flow of HQMR. Through various forms of medical consortia, the government could cultivate a number of medical groups with obvious brand advantages and provide high-level services across regions, and promote the grouping and branding of HQMR [13]. Third, the full use of "Internet + Medical", artificial intelligence, big data, telemedicine, and other advanced technologies expands the service scope of advantageous medical resources [52]. Internet hospitals offer convenient outpatient delivery regardless of the patients’ distance from the hospital [53]. In certain high-income countries such as the United Kingdom, the United States, Japan, the use of the Internet for video consultation or health advice for patients has helped alleviate the shortage of health resources to some extent [54, 55]. Some research shows that artificial intelligence technologies, such as virtual AI and telemedicine, are expected to help China overcome current limitations in the allocation of healthcare resources and alleviate the pressures associated with access to high-quality medical care [56].

This study has several limitations. Firstly, the distribution of HQMR is measured by the number of high-level hospitals, without considering the number of beds and medical personnel in high-level hospitals. In addition, in the demonstration of the influencing factors, only data on social and economic aspects in 2020 were used, hence the trend of the influence of factors on HQMR cannot be fully demonstrated. Finally, because of the limited data availability, we only discussed the distribution of HQMR at the provincial level, and the distribution of HQMR at the prefecture cities and counties levels needs to be further explored in future studies.

## Conclusion

China's total HQMR is growing rapidly but is relatively inadequate. The distribution of HQMR by population is better than by geography, and the distribution by geography is less equitable. Population size and geographical area both need to be taken into account when formulating policies, rather than simply increasing the number of HQMR. In order to improve the access of all citizens to high quality medical services, it is recommended to accelerate the expansion and balanced layout of HQMR and promote coordinated regional development, rather than simply increasing the number of HQMR.

## Data Availability

The survey data collected and analyzed during the current study are available from the corresponding author on reasonable request.

## Abbreviations

CNY: Chinese yuan
HHRDI: High-quality health resource density index
HQMR: High quality medical resources
G: The Gini coefficient
T: Theil Index
GDP: Gross Domestic Product
GRs: The growth rates
